# PMMA Solution Assisted Room Temperature Bonding for PMMA–PC Hybrid Devices

**DOI:** 10.3390/mi8090284

**Published:** 2017-09-20

**Authors:** In-Hyouk Song, Taehyun Park

**Affiliations:** 1 Department of Engineering Technology, Texas State University, San Marcos, TX 78666, USA; 2 School of Mechanical Engineering, Kyungnam University, Changwon 51767, Korea

**Keywords:** polymethyl methacrylate (PMMA) spin coated polycarbonate (PC), microfluidic, hybrid bonding, thermal bonding, PMMA–PC bonding

## Abstract

Recently, thermoplastic polymers have become popular materials for microfluidic chips due to their easy fabrication and low cost. A polymer based microfluidic device can be formed in various fabrication techniques such as laser machining, injection molding, and hot embossing. A new bonding process presented in this paper uses a 2.5% (*w*/*w*) polymethyl methacrylate (PMMA) solution as an adhesive layer to bond dissimilar polymers—PMMA to polycarbonate (PC)—to enclose the PMMA microfluidic channels with PC. This technique has been successfully demonstrated to bond PMMA microchip to PC film. This paper presents bonding strength using a shear strength test and a crack opening method in addition to the fluidic leakage inspection.

## 1. Introduction

Microfluidic systems for bio-/chemical analysis have become considerably attractive due to fast analysis, small reagent consumption, on-chip sample treatment, and portability [1]. The microfluidic devices have been demonstrated using not only glass or silicon wafer but also polymer, which has superior disposability, ease of fabrication, and a lower cost [2]. In addition, polymer based microdevices offer the advantages of biocompatibility, flexibility in manufacturing, and applicability in mass production. The most popular thermoplastic polymers for microfluidic devices are polymethyl methacrylate (PMMA), polycarbonate (PC), cyclic olefin copolymer (COC), and polystyrene (PS) [3,4,5,6].

Followed by microfluidic channel patterning on thermoplastic polymer, a sealing process is required on the formed polymer to enclose microfluidic channels. The sealing process is one of crucial but challenging steps to complete polymer-based microfluidic devices and needs to enclose the channel tightly without deforming the microchannels. The bonding techniques for sealing microfluidic channels or chambers require robust sealing but are faster and simpler without channel deformation and clogging. Various bonding techniques have been reported using direct or indirect bonding techniques, such as surface modification [7,8,9,10,11], chemical gluing [12,13,14], thermoplastic fusion bonding [7,11,15,16,17], adhesive bonding [18,19,20], and ultrasonic bonding [21]. 

The proposed research presents a new technique for bonding dissimilar polymers—PMMA and PC—for microfluidic devices. PMMA is one of the most popular materials in polymer based microfluidic devices due to easy fabrication and PC is used in various applications for its outstanding properties and especially high toughness, high impact strength, and high optical transparency [22]. However, it is impossible to bond PMMA to PC directly using a thermoplastic fusion bonding because of the different glass transition temperatures. In this paper, PMMA solution is employed as an adhesive layer to bond PMMA to PC. The former contains microfluidic channels and the latter is used for a covering material of the microfluidic device. The proposed bonding process is performed at room temperature and low compression force for bonding. The details of the fabrication process and the bonding strength tests are presented in this paper.

## 2. Fabrication and Bonding of PMMA–PC

**Figure 1:**
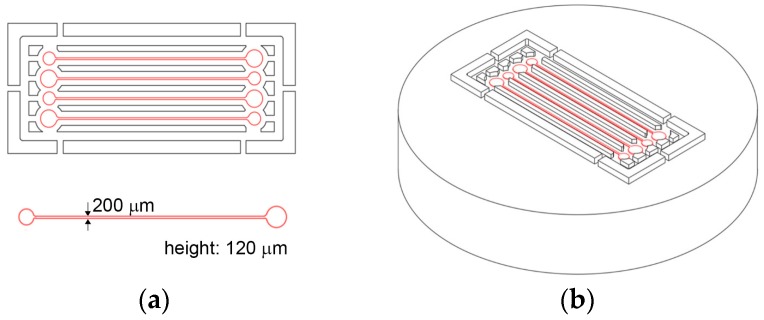
(**a**) Design of microfluidic channels. Channel width and height are 200 µm and 120 µm, respectively. (**b**) 3D illustration of the master mold design containing four microfluidic channels.

Hot embossing uses a micromachined mold insert to transfer microfluidic channels embedded in mold onto polymer substrate. Figure 1a shows the design of the master mold containing four microfluidic channels placed in parallel. The channel width and height are designed of 200 µm and 120 µm, respectively. Figure 1b illustrates 3D image of the designed master mold. A commercial CNC machining center was used to form a brass mold insert. Microfluidic channels are replicated on 3 mm thick plane PMMA sheet, which is annealed in an oven at 80 °C. The embossing is accomplished with a hot press (QM900M, QMESYS, Gunpo-si, Gyeonggi-do, Korea). The PMMA sheet including mold insert is placed onto the lower platen of the hot press, which is configured to maintain 160 °C and slowly fastened with a pressure of 8 MPa for 8 min. Upon completion of the holding time, PMMA is cooled down and separated from the mold insert at 80 °C.

**Figure 2:**
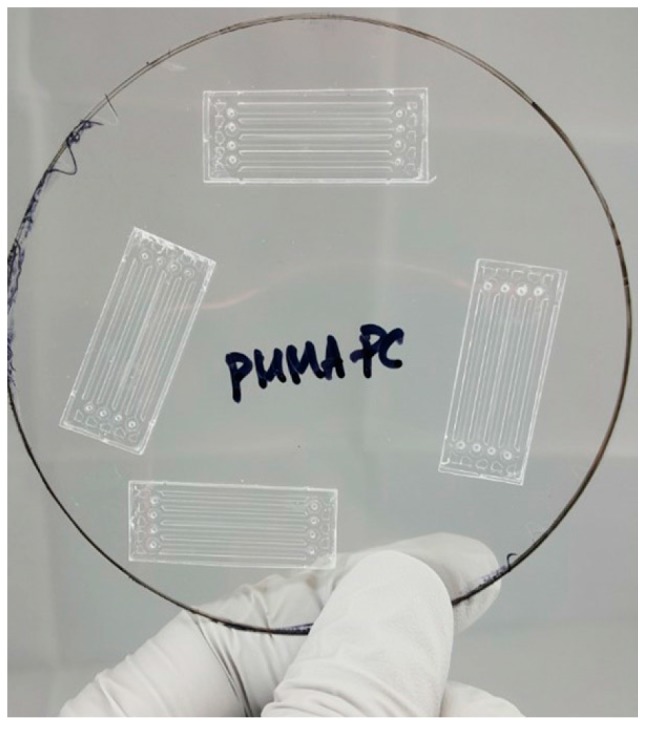
Bonded PMMA microchips on PSC-PC film.

After hot embossing, the microfluidic channels in PMMA microchips are enclosed with a 250 µm thick PC film using an adhesive layer. The adhesive layer is, in this research, used with a 2.5% (*w*/*w*) PMMA solution dissolved in propylene glycol monomethyl ether acetate (PGMEA, CAS# 108-65-6, Sigma-Aldrich, Inc., St. Louis, MO, USA). The PMMA solution is made by dissolving 7.5 g of PMMA beads (molecular weight: 75,000, Polysciences, Inc., Warrington, PA, USA) in 300 g of PGMEA. Once PMMA beads are properly dissolved in the PGMEA, PMMA solution is filtered with a 0.2 μm membrane syringe filter and spin-coated on a PC sheet. Three steps of spin coating angular velocity are used. The first step is 500 rpm for 5 s, the second step is 4500 rpm for 25 s, and the last step is 1000 rpm for 5 s. The thickness of the spin-coated 2.5% PMMA solution is less than 100 nm at 4500 rpm. Right after taking out the PMMA spin coated PC called PSC-PC film, the hot embossed PMMA chip is placed face-down on PSC-PC film and sealed using a hand roller at room temperature before the PMMA solution is completely dried. Figure 2 shows the bonded PMMA microchips on PSC-PC film.

## 3. Characterization of Bonding 

### 3.1. Microchannel Inspection

**Figure 3:**
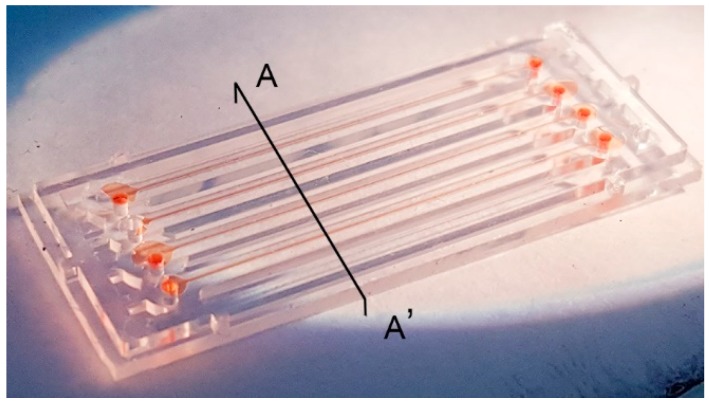
Leakage test of (PMMA)–PC microchip with red food dye.

The aim of this study is to demonstrate the usefulness of PMMA solution as an adhesive layer for bonding PMMA microchip to PC film. The bonding quality of the fabricated microfluidic chips is investigated by observation of leakage across the bonded interface. Red food dye is passed into the fabricated microfluidic channels of Figure 2. As shown in Figure 3, all four channels are filled with red food dye after removing capillary connection at the drain reservoir without leakage at any point along the microchannels.

**Figure 4:**
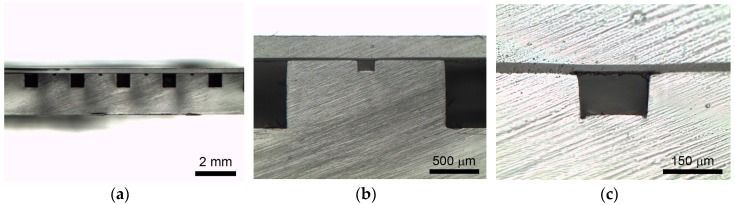
(**a**) Cross-sectional micrograph of the PMMA–PC microchip. (**b**) Close-up view of Figure 4a. (**c**) Closed-up view of the cross-sectional microchannel of Figure 4b.

Channel deformation is a common issue in using a thermoplastic fusion bonding technique since the Young’s modulus of viscoelastic materials dramatically change with relatively small changes in temperature in the glass transition region. Besides, thermoplastic fusion bonding cannot guarantee an even pressure distribution over the contact area or precise temperature control. In contrast, the use of a 2.5% (*w*/*w*) PMMA solution can avoid channel deformation since the process is carried out at room temperature. Figure 4 shows the cross-sectional view of microfluidic chips along the line of A-A’ in Figure 3. The boundary of the microfluidic channel walls are sufficiently clear without deformation. In contrast to PMMA, PC is not dissolved by propylene glycol monomethyl ether acetate (PGMEA) immediately. However, it is observed that PC tends to absorb the solution, and this induces softening in the surface of PC. The softened PC with the dissolved PMMA makes the surface sticky and improved the bonding strength between PC film and PMMA microchips. A dark band of PC film surface contacting with the PMMA microchip is seen in Figure 4c, which results from the penetration of the dissolved PMMA solution into a PC film during spin coating and bonding. 

### 3.2. Bonding Strength

**Figure 5:**
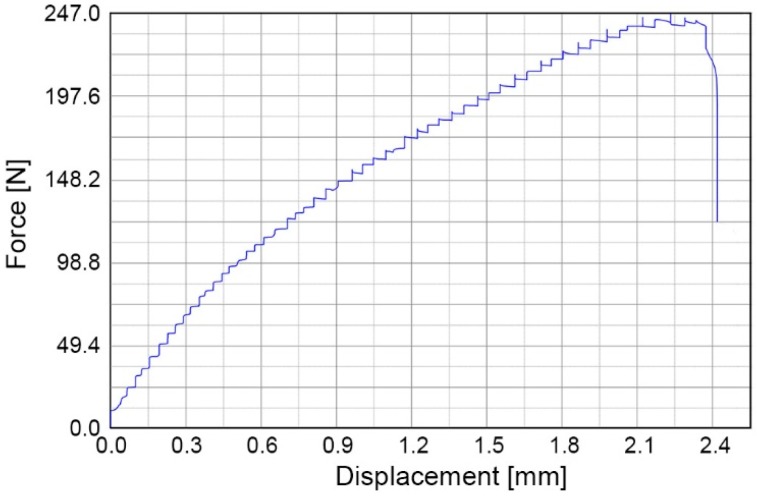
Loading cycle of shear strength test of PMMA–PC microchips.

The bonding strength is tested using two methods: (1) shear strength test and (2) crack opening method. The shear strength of the bonded PMMA–PC chips is tested with a universal testing machine (DR-100, DR-TECH, Grantsburg, WI, USA), which is useful for testing thin bonded specimens. For each specimen, PMMA is bonded to 250 µm thick PSC-PC film with an overlapping bond area (20 mm × 17.4 mm). Shear force is applied to the specimen through a wedge at the PMMA–PC interface at a crosshead speed of 10 mm/min until the bonded chip is delaminated. Figure 5 shows a loading cycle where the pulling force increases with the elongation of the bonded PMMA–PC chip until failure. Three specimens are tested at room temperature and the average load at failure of the bonding is 0.721 ± 0.03 MPa.

**Figure 6:**
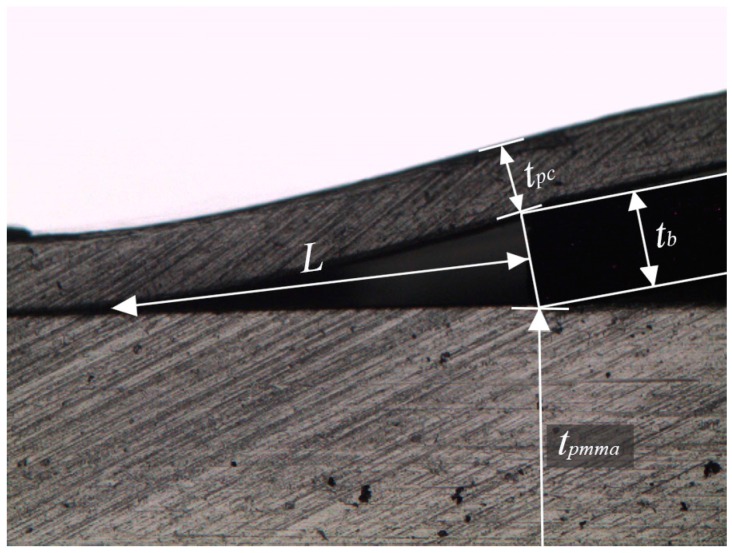
Crack opening method for measuring the bonding strength.

After the bonding process, the bonding strength is determined using a crack opening method [23]. A blade is inserted interface at the bonded PMMA–PC polymers. The bonding strength is then calculated using

(1) $$\gamma =\frac{3}{16\cdot L^{4}}\cdot \frac{t_{b}^{2}}{\left(\frac{1}{E_{pc}\cdot t_{pc}^{3}}+\frac{1}{E_{pmma}\cdot t_{pmma}^{3}}\right)}$$

where γ (J/m^2^) is the bonding strength. *E_pc_* (=2–2.44 GPa) and *E_pmma_* (=2.24–3.8 GPa) are the Young’s modulus of the PC and PMMA, respectively [24]. The thicknesses of the PC sheet, *t_pc_*, and the PMMA microchip, *t_pmma_*, are measured of 220 µm and 2.122 mm, respectively. The blade thickness, *t_b_*, is of 353 µm. The length of the crack, *L*, is measured as shown in Figure 6. Four specimens are measured and the bonding strength is calculated of 126 ± 19 J/m^2^ for *E_pc_* (=2 GPa) and *E_pmma_* (=2.24 GPa), which shows more than 50 times higher bonding strength than the previous reported hybrid thermoplastic to thermoplastic (PMMA–PS) bonding using a thermocompression method [25].

Additional bonding test is carried out by letting compressed air pass through the microfluidic channels and the pressures are measured at inlet and at the outlet of the microfluidic channels. The channels are able to withhold 75 psi at outlet and 95 psi at inlet without delamination, which is the maximum supplied pressure from the air compressor. 

## 4. Conclusions

In this study, a simple and fast bonding technique for fabrication of PMMA–PC hybrid devices is developed using assistance of PMMA solution made by dissolving PMMA beads in PGMEA solution. This technique uses only PMMA solution as an adhesive layer and all process are performed at room temperature. PMMA–PC microfluidic chips are successfully bonded with high bonding strength and low dimension loss using the proposed method. The values for the bonding strength are tested by a shear strength test and a crack opening method of 0.721 ± 0.03 MPa and 126 ± 19 J/m^2^, respectively. According to a burst test, the channels withstand more than 75 psi without delamination. Moreover, the proposed technique is feasible for bonding multiple devices on the same substrate.
